# Adult height, body mass index change, and body shape change in relation to esophageal squamous cell carcinoma risk: A population‐based case‐control study in China

**DOI:** 10.1002/cam4.2444

**Published:** 2019-08-01

**Authors:** Xiaorong Yang, Tongchao Zhang, Xiaolin Yin, Ziyu Yuan, Hui Chen, Amelie Plymoth, Li Jin, Xingdong Chen, Ming Lu, Weimin Ye

**Affiliations:** ^1^ Clinical Epidemiology Unit Qilu Hospital of Shandong University Jinan China; ^2^ Department of Epidemiology and Health Statistics, School of Public Health Shandong University Jinan China; ^3^ Fudan University Taizhou Institute of Health Sciences Taizhou China; ^4^ Department of Medical Epidemiology and Biostatistics Karolinska Institutet Stockholm Sweden; ^5^ State Key Laboratory of Genetic Engineering, Collaborative Innovation Center for Genetics and Development, School of Life Sciences Fudan University Shanghai China

**Keywords:** body mass index, body shape, body size change, esophageal squamous cell carcinoma, height, risk factor

## Abstract

The relationship between risk of esophageal squamous cell carcinoma (ESCC) and adult height, changes in individual body mass index (BMI) and body shape is not established. We performed a large population‐based case‐control study, which enrolled a total of 1414 ESCC cases and 1989 controls in a high‐incidence area in China. Using face‐to‐face interview with a structured questionnaire, information on participants' heights, weights, and perceived body shapes at 20 years of age was collected. Additionally, data on weight and perceived body shape among the same participants 10 years prior to ascertainment were collected using the same method. Odd ratios (ORs) of ESCC risk in relation to BMI and body shape were estimated using unconditional logistic regression models. The adjusted results indicated that ESCC risk in adults rapidly rose as height increased, plateauing at 170 cm among men and 157 cm among women. Among participants who were underweight, normal weight, or thinner than body shape 4, body weight loss was associated with increased risk of ESCC, and body weight gain was associated with decreased incidence of ESCC (ORs ranging from 0.40 to 0.76). Notably, however, changes in body weight did not significantly affect ESCC risk among participants who were overweight, obese, or larger than body shape 3. Maintaining a fit body shape and a reasonable BMI is advisable and of vital importance to reduce the risk of ESCC, especially in high‐risk areas.

## 1 INTRODUCTION

According to the International Agency for Research on Cancer (IARC), about 572 000 new esophageal cancer cases (3.2% of all cancers) and 509 000 deaths from esophageal cancer (5.3% of all cancer deaths) were reported worldwide in 2018.^1^ Around half of all new esophageal cancer cases occurred in China.^2^ Esophageal cancer has two predominant histopathological subtypes: esophageal squamous cell carcinoma (ESCC) and esophageal adenocarcinoma (EAC). The risk factors and genomic characteristics of each of these cancers are quite different.^3^, ^4^ Most studies show that the risk of EAC is inversely associated with adult height,^5^, ^6^, ^7^ but the associations between ESCC risk and adult height are erratic.^6^, ^7^ It is well documented that higher body mass index (BMI) is a significant risk factor for many diseases, including many types of cancer.^8^ Nevertheless, with the increase of BMI, contrasting risk association patterns for ESCC and EAC are typically observed: elevated BMI has been shown to be associated with increased incidence of EAC,^9^, ^10^, ^11^, ^12^, ^13^ and most studies including a meta‐analysis indicate that BMI is inversely associated with ESCC development.^14^, ^15^, ^16^, ^17^ However, weight loss may be a consequence of late‐stage cancer syndromes.

Although body weight is a changeable factor across adulthood, limited number of studies have considered the relationship between weight change during adulthood and ESCC incidence, except reports from Lahmann et al and Chow et al which pointed out that weight gain greater than 20 kg during adulthood is associated with approximately 50% decreased risk of ESCC.^14^, ^18^ No similar association was observed by Merry et al.^6^ Considering that equivalent changes in body weight based on different baseline BMI status (eg, lean and obese) may have distinct influences on health, the stratified analysis needs be further explored.

In addition to BMI, perceived body shape as estimated using Stunkard's Figure Rating Scale can also be applied to reflect different body sizes, especially for previous body somatotypes,^19^ in situations where some specific measurements are unavailable (eg, waist‐hip ratio). In an American cohort study, increasingly large perceived body shape at any point in life was found to be associated with a higher risk of EAC in males (relative risk ranged from 1.23 to 3.01).^20^ However, the relationship between perceived body shape and ESCC risk has not yet been explored. The application of body shape will expound the relationship between body shape and ESCC occurrence in another way, and may provide further support for the relationship between BMI change and ESCC risk.

We initiated a rigorously designed population‐based case‐control study of upper gastrointestinal cancer in Taixing, a high‐incidence area in China.^21^ To reduce potential report bias, we did our best to collect questionnaire data before the cases were aware of disease diagnosis. The progression time from symptomatic precancerous lesions to ESCC diagnosis was not yet established. Based on clinical prior knowledge, body size is unlikely to be altered due to early symptoms 10 years preceding the diagnosis of ESCC. Since individual body sizes in most Chinese adults usually monotonically change with age, the body sizes at age 20 years and at 10 years prior to interview were used to reflect the status at early adulthood, and at advanced age before cancerous symptoms might cause changes. Because more than 95% of esophageal cancer cases are ESCC in China,^22^ we focus in this analysis on the effects of adult height, BMI, and perceived body shape change on the risk of ESCC in this high‐incidence Chinese population.

## 2 MATERIALS AND METHODS

### 2.1 Study design and participants

Details of the research design and the selection flow of participants have previously been described.^23^, ^24^ In short, we performed a case‐control study in Taixing, China, in which 40‐85 year‐old participants who had lived in Taixing for at least 5 years were recruited. We attempted to enroll all newly diagnosed esophageal cancer cases from October 2010 to September 2013 from four of the largest local hospitals (covering almost 90% of local clinical diagnoses). Once doctors suspected that patients might have esophageal cancer during endoscopy examination, these patients were asked to complete a questionnaire by trained interviewers. After histopathological examination, patients who were not histopathologically confirmed were excluded from the study. To further replenish esophageal cancer cases missed in the endoscopy units for various reasons, linkages and reconciliation with the local Cancer Registry were conducted at the end of each year.

During the three‐year period, we gathered 1401 suspected cases from the hospitals’ endoscopy units and added 280 reported cases via the local Cancer Registry. For each case, we attempted to collect sections from formalin‐fixed and paraffin‐embedded tissue blocks and original pathological reports after surgical resection. In summary, we gathered tissue sections from 1499 suspected or reported cases, and original surgical pathological records for 83 cases among the remaining 182 cases for whom tissue blocks were not available. The remaining 99 nonoperated cases were excluded from our study. After the pathological sections were reviewed by the study pathologist and surgical pathological reports were reassessed, a total of 1499 esophageal cancer cases (1418 cases of ESCC and 81 cases of non‐ESCC) were eligible for inclusion and were thus enrolled into the study (participation rate: 78.3%).

During the same period, to increase the statistical power of the study, we employed a frequency‐matched method for selection of control subjects, where the strata were defined by sex and 5‐year age groups. For each stratum of the cases of upper gastrointestinal cancer, we selected corresponding controls from the local Population Registry every year with a 1.3:1 ratio, considering an approximately 75% response rate among controls based on our pilot study. Finally, 1992 eligible controls participated in the current study (participation rate: 70.4%).

The current analysis was based on the 1418 ESCC cases, which were independently reviewed and confirmed, and 1992 controls. After further excluding seven subjects who had incomplete questionnaire information on the main exposure variables, we included 1414 cases and 1989 controls in the final analysis.

### 2.2 Ethics statement

The study protocol was approved by the Institutional Review Boards of the School of Life Sciences, Fudan University (date: 19 February 2009) and Qilu Hospital, Shandong University (date: 8 March 2010). The study was carried out in accordance with the approved protocol, and all participants provided written informed consent. The raw data and analysis code of this study are available from the corresponding authors on reasonable request.

### 2.3 Exposure assessment

All participants underwent face‐to‐face interviews with trained staff using an electronic structured questionnaire. The questionnaire covers information on demographics (including height, weight and perceived body shape at 20 years of age, weight and perceived body shape 10 years prior to ascertainment), socioeconomic status, family history of cancer, oral hygiene, personal medical history, smoking, alcohol and tea drinking history. For the collection of perceived body shape information, the trained staff presented and briefly introduced the electronic image of the revised Stunkard Graphic Rating Scale (Figure S1) and then asked participants to choose the closest perceived body shape at age 20 years and 10 years ago.

Adult height was estimated by height at 20 years of age and was considered a stable variable in adulthood.^25^ BMI status was categorized based on Chinese standards for BMI thresholds (unit: kg/m^2^): underweight (<18.5), normal (≥18.5 and <24), overweight (≥24 and <28), and obese (≥28). Perceived body shape was assessed using the revised Stunkard's Figure Rating Scale (Figure S1),^26^, ^27^ which illustrates seven male and nine female visible schematic silhouettes, ranging from extreme thinness (body shape 1 is leanest) to extreme adiposity (body shape 7 is largest for males and body shape 9 is largest for females).

### 2.4 Statistical methods

We used restricted cubic spline regression models with five knots to estimate the nonlinear relationship of adult height, BMI at age 20 years, and BMI 10 years prior to ascertainment with the risk of ESCC. For BMI and perceived body shape at different stages and changes in BMI status and perceived body shape, we applied unconditional logistic regression models to calculate odds ratios (ORs) with 95% confidence intervals (CIs) for ESCC risk. The categories with the largest sample size, namely the normal BMI group and perceived body shape 3, were used as the reference, and some categories with only a few subjects were merged into adjacent categories. For the fully adjusted regression models, we adjusted for age (continuous), sex, education (illiteracy/primary school/junior high school/high school or above), marital status (unmarried/married/divorced or widowed), occupation (farmer/worker/other), family wealth score (quintiles), missing and filled teeth (none/ <6/ ≥6), daily frequency of brushing teeth (<2/ ≥2), tea temperature (never/warm/hot/very hot), family history of esophageal cancer among first‐degree relatives (yes/no), smoking pack‐years (never/ ≤30/ >30) and alcohol drinking intensity (never/ ≤80/ >80 g/day). Selection of these covariates was based on the criterion that there existed statistically significant correlations between body composition and these potential covariates. The family wealth score was calculated based on ownership of some valuable home items using multiple correspondence analysis and categorized as quintiles according to the observed coordinates among control participants.^28^ To evaluate effect modification on the associations between BMI and perceived body shape with ESCC risk by sex, family wealth score, smoking, and alcohol drinking, we used likelihood ratio tests for nested models with and without interaction terms to obtain *P* values for interaction. All analyses were carried out using STATA version 13.1 (Stata Corporation). Two‐sided *P* values less than .05 were considered statistically significant.

## 3 RESULTS

The characteristics of 1989 control subjects and 1414 ESCC cases are summarized and presented in Table S1. The matching variables for age and sex were equivalent between ESCC cases and controls. The ESCC cases tended to have lower education level, lower family wealth score, more missing and filled teeth, fewer times of tooth brushing per day, drink hotter tea beverage, consume more tobacco and alcohol, and were more likely to have a family history of esophageal cancer among first‐degree relatives.

Table 1 presents the general distribution of height at 20 years of age, BMI at 20 years of age, BMI 10 years prior to ascertainment, Stunkard body shape at 20 years of age, and Stunkard body shape 10 years prior to ascertainment among controls and ESCC cases. Both male and female cases were on average taller than controls (*P* < .001 and about 3 cm difference in both males and females). BMI status and perceived body shape at 20 years of age in ESCC cases were equivalent to controls. However, ESCC cases tended to have a much leaner BMI status and Stunkard body shape 10 years prior to ascertainment compared to controls.

**Table 1:** Height, BMI, and body shape characteristics in a population‐based case‐control study of esophageal squamous cell carcinoma in Taixing, China, 2010‐2013

Anthropometric measure	Controls (N = 1989) N (%)	Cases (N = 1414) N (%)	*P* value^a^
Height (cm) at 20 y of age (mean ± SD)			
Males (N = 2230)	164.8 ± 7.0	167.9 ± 5.9	<.001
Females (N = 1073)	154.3 ± 6.2	157.3 ± 5.0	<.001
BMI status (kg/m^2^) at 20 y of age			
<18.5 (Underweight)	221 (11.11)	141 (9.97)	.769
18.5‐24 (Normal)	1343 (67.52)	968 (68.46)	
24‐28 (Overweight)	375 (18.85)	269 (19.02)	
≥28 (Obese)	50 (2.51)	36 (2.55)	
BMI status (kg/m^2^) 10 y prior to ascertainment			
<18.5 (Underweight)	110 (5.53)	132 (9.34)	<.001
18.5‐24 (Normal)	1205 (60.58)	903 (63.86)	
24‐28 (Overweight)	545 (27.40)	322 (22.77)	
≥28 (Obese)	129 (6.49)	57 (4.03)	
Perceived body shape at 20 y of age			
Shape 1	110 (5.53)	88 (6.22)	.743
Shape 2	458 (23.03)	347 (24.54)	
Shape 3	744 (37.41)	523 (36.99)	
Shape 4	496 (24.94)	335 (23.69)	
Shape 5	143 (7.19)	95 (6.72)	
Shape 6	32 (1.61)	19 (1.34)	
Shape 7/8/9	6 (0.30)	7 (0.50)	
Perceived body shape 10 y prior to ascertainment			
Shape 1	50 (2.51)	101 (7.14)	<.001
Shape 2	318 (15.99)	308 (21.78)	
Shape 3	695 (34.94)	470 (33.24)	
Shape 4	587 (29.51)	343 (24.26)	
Shape 5	243 (12.22)	148 (10.47)	
Shape 6	74 (3.72)	35 (2.48)	
Shape 7/8/9	22 (1.11)	9 (0.64)	

Abbreviations: BMI, body mass index; N, number; SD, standard deviation.

a *P* values were derived using Wilcoxon rank‐sum test for continuous variables and Chi‐squared test for categorical variables.

**Figure 1:**
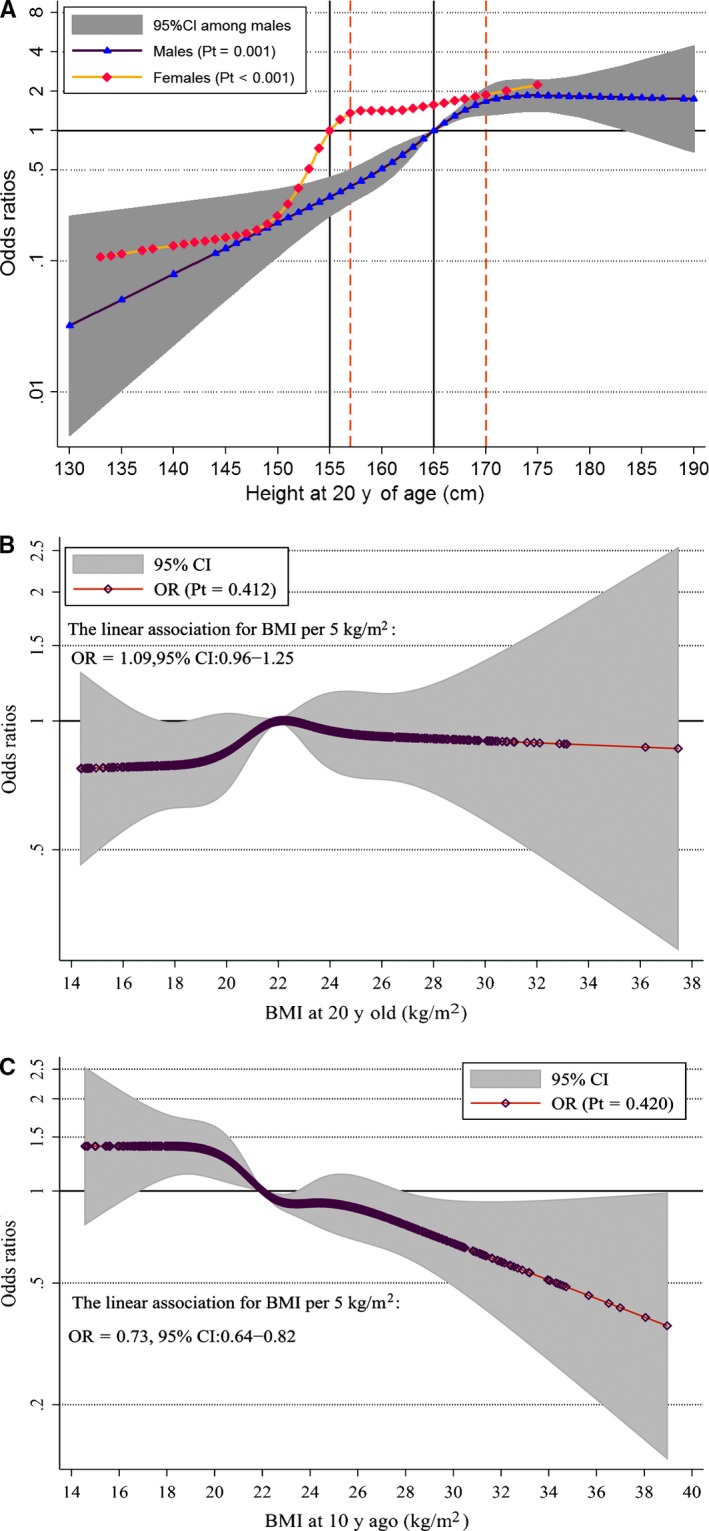
The nonlinear association between adult height, BMI at different y and the risk of esophageal squamous cell carcinoma (ESCC). A, Adult height at 20 y of age among men (purple) and women (gold), respectively. B, BMI at 20 y of age. C, BMI 10 y prior to ascertainment. This red curve presents the OR values estimated by the restricted cubic spline regression model. The dot indicates that there is at least one actual value of the X axis. Pt is the *P* value testing for departure from linearity

Figure 1A shows the positive nonlinear relationship between height at age 20 years and ESCC risk (male reference: 165 cm; female reference: 155 cm), after adjusting for potential confounders; the *P* values testing for departure from linearity were .001 in males and less than .001 in females, respectively. Before the adult height reached 170 cm among men and 157 cm among women, ESCC risk increased sharply by about 25% for every 5 cm increase in height, and this ascending trend of ESCC risk then somewhat plateaued; similar trends were found for categorized height as shown in Table S2. Linear trends between ESCC risk and BMI at age 20 and BMI 10 years prior to ascertainment were detected (Figure 1B and Figure 1C); an increase in BMI 10 years prior to ascertainment was associated with a linear decrease in the risk of ESCC (OR = 0.73, 95% CI = 0.64‐0.82 for BMI per 5 kg/m^2^ increase), but no significant association between ESCC risk and BMI at 20 years of age was found (OR = 1.09, 95% CI = 0.96‐1.25 for BMI per 5 kg/m^2^ increase, Table S2).

**Figure 2:**
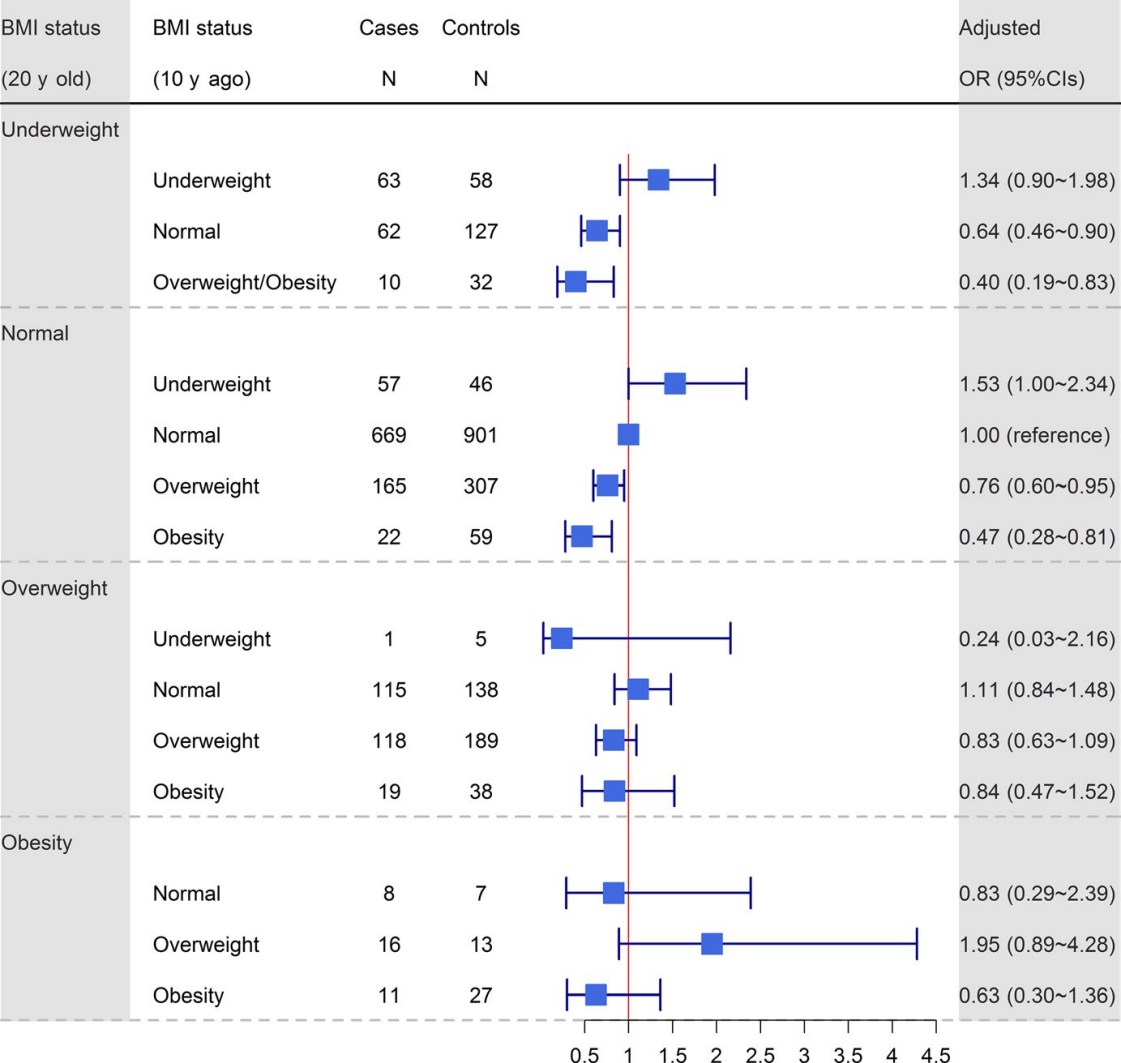
The association between BMI status change from 20 y of age to 10 y prior to ascertainment and the risk of esophageal squamous cell carcinoma (ESCC). The black square indicates the OR of each category. The horizontal line represents the 95% CI

Figure 2 illustrates the association between ESCC risk and BMI status change from age 20 years to 10 years prior to ascertainment. For underweight and normal weight participants, decreasing BMI status was associated with increased ESCC risk (compared with unchanged normal BMI status from ages 20 to 10 years prior to ascertainment, the OR of the change from normal weight to lean weight was 1.53, 95% CI: 1.00~2.34) and increasing BMI status was associated with decreased ESCC risk (ORs ranging from 0.30 to 0.76). However, decreasing or increasing BMI status did not significantly change the ESCC risk among overweight and obese participants. Similar trends were observed when stratifying participants by age at interview (no more than 65 years old vs more than 65 years old). However, most of the observed associations were more evident in the older group than in the younger group (Figure S2).

**Figure 3:**
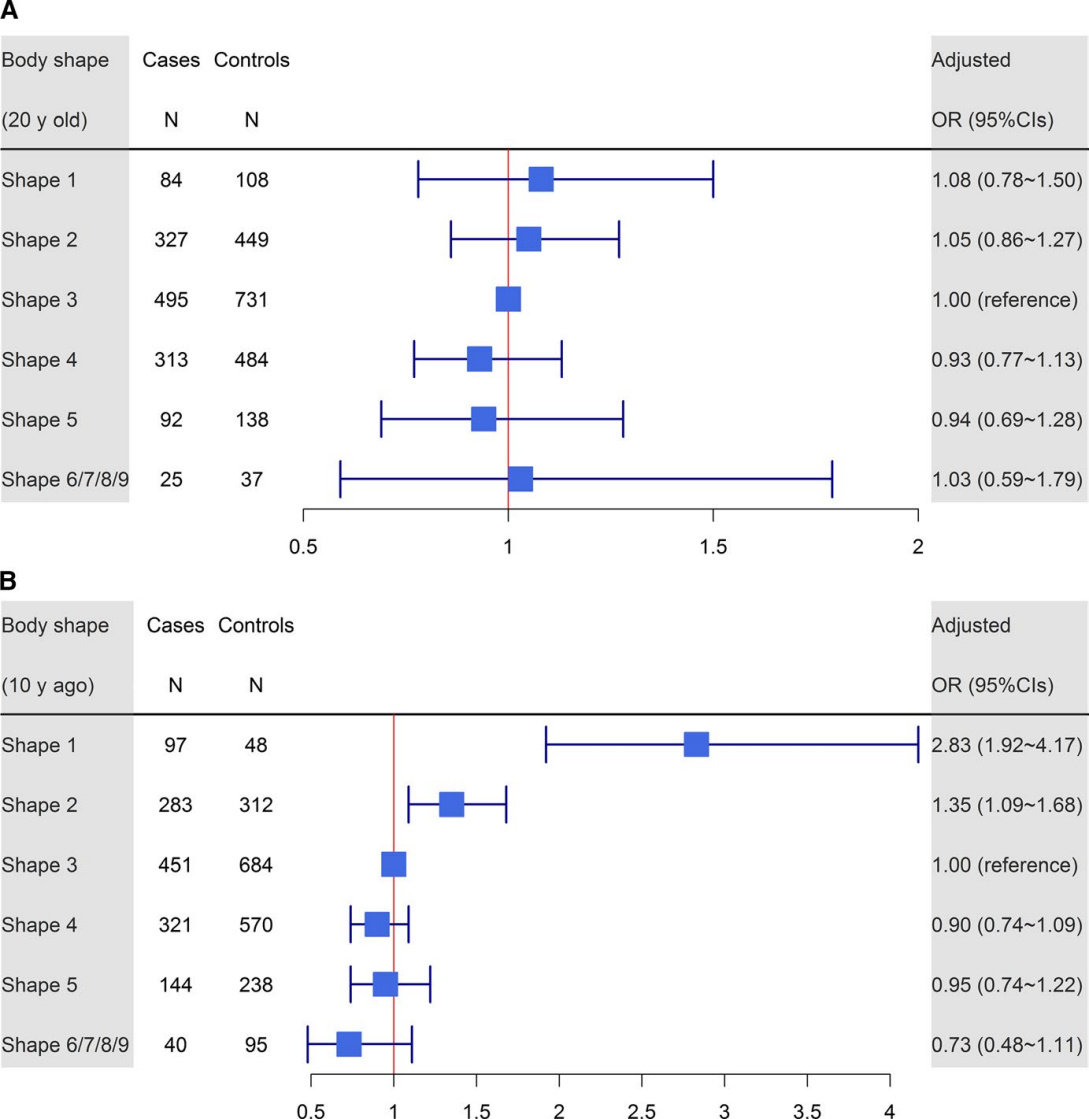
The association between perceived body shape at different y and the risk of esophageal squamous cell carcinoma (ESCC). A, Body shape at 20 y of age. B, Body shape 10 y prior to ascertainment. The black square indicates the OR of each category. The horizontal line represents the 95% CI

Figure 3 presents the associations between Stunkard body shape at different years and the risk of ESCC. Perceived body shape at 20 years of age was not associated with ESCC risk (*P* value for trend .276, Figure 3A). However, participants with body shape 1 and 2, 10 years prior to ascertainment, had higher risks of ESCC than those with other body shape types (compared with body shape 3, the OR of body shape 1 was 2.83, 95% CI: 1.92~4.17, P value for trend less than 0.001, Figure 3B).

**Figure 4:**
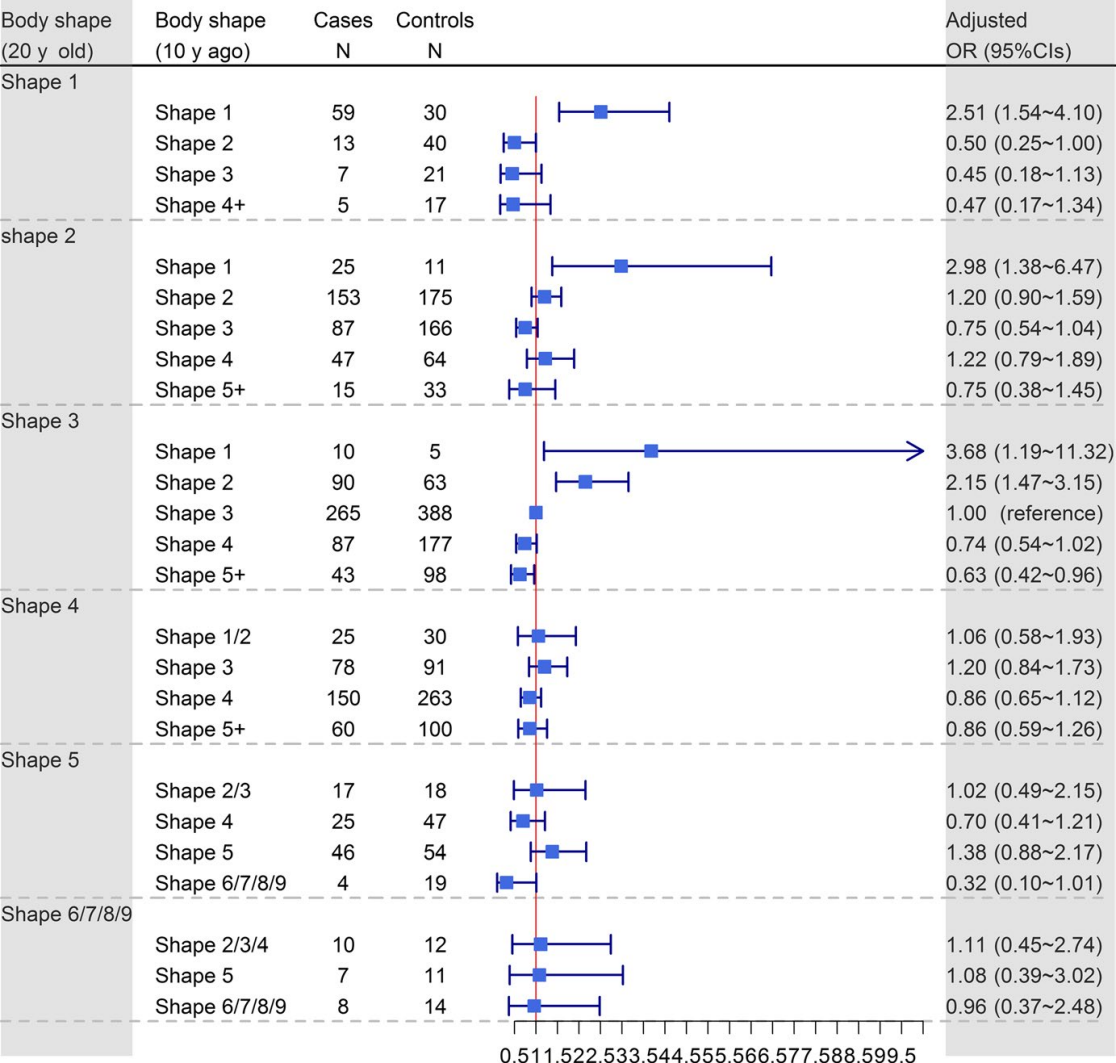
The association between perceived body shape change from 20 y of age until 10 y prior to ascertainment and the risk of esophageal squamous cell carcinoma (ESCC). The black square indicates the OR of each category. The horizontal line represents the 95% CI

Figure 4 summarizes the relationship of ESCC risk with Stunkard body shape change from 20 years of age until 10 years prior to ascertainment. A decrease in body shape rating was associated with a greater than twofold risk of ESCC, and an increase in body shape rating was associated with decreased ESCC risk (most ORs were less than 1.00) among those with body shape ratings of 1, 2, or 3 at age 20. However, body shape change did not significantly affect ESCC risk among participants with body shape rating greater than 3 at age 20.

We further examined the potential effect of modification of sex, family wealth, smoking, or alcohol drinking on the associations between BMI status change, perceived body shape change, and ESCC risk. The results showed that the ESCC risk change trend was similar (data not shown), and the likelihood ratio test suggested that there was no significant interaction (all *P* values of interaction >.05).

We conducted a sensitivity analysis by excluding 161 cases added from the local Cancer Registry. The overall results did not change substantially (data not shown).

## 4 DISCUSSION

From this large population‐based case‐control study in a high‐risk Chinese population, we found that the risk of ESCC in adults rose steadily with increasing height up to 170 cm among men and 157 cm among women, after which the risk of ESCC somewhat plateaued. Although BMI and perceived body shape at 20 years of age were not associated with ESCC risk, BMI and perceived body shape 10 years prior to ascertainment showed an inverse linear relationship with ESCC risk. Among participants who were underweight, normal weight, or leaner than perceived body shape 4, loss of body weight was associated with increased ESCC risk while gain of body weight was associated with decreased ESCC risk. However, change in body size did not significantly affect ESCC risk among participants who were overweight, obese, or rated larger than perceived body shape 3.

Engeland et al^7^ stated that ESCC risk inversely correlates with adult height in men, but not in women. Moreover, Merry et al and Steffen et al did not observe any significant association, likely due to limited sample size.^6^, ^29^ With the help of the large sample size in our study, we identified nonlinear and monotonic positively increasing associations between adult height and ESCC risk for both males and females. Multiple cohort studies reported that adult height is positively associated with risks of all‐site cancers and cancer‐related deaths, but they did not identify the association between height and ESCC risk.^30^, ^31^, ^32^

The evidence for low BMI status or thinness as a risk factor for ESCC has been documented over the recent decades. Smith et al performed a meta‐analysis indicating that each 5 kg/m^2^ increase in BMI is associated with a 51% (95% CI: 45%‐56%) and 31% (95% CI: 25%‐37%) lower ESCC risk in case‐control studies and prospective studies, respectively.^17^ Our results indicate that BMI at age 20 years is not significantly associated with ESCC incidence, which is consistent with two previous studies,^6^, ^14^ but the risk of ESCC decreases linearly with BMI increase 10 years prior to ascertainment. The decreasing trend of ESCC risk in relation to BMI is in line with the summary estimate of risk reduction from the above‐mentioned meta‐analysis.^17^ Two studies reported that individual BMI gain is associated with a lower risk of ESCC than maintaining a steady BMI during adult life.^14^, ^18^ Nevertheless, the same absolute body weight gain among lean or obese participants may have different health influences; thus we analyzed the risk of ESCC in relation to change in individual BMI status from 20 years of age to 10 years prior to ascertainment by baseline BMI status. Our data suggest that decreasing BMI status is associated with increased ESCC risk, while increasing BMI status is associated with decreased ESCC risk among originally underweight and normal weight participants. However, individual body weight change was not significantly associated with ESCC incidence among originally overweight and obese participants. Song et al also pointed out that an increase in BMI during adulthood decreased the risk of developing ESCC among nonoverweight subjects using a Japanese cohort study, although the result was not robust due to a relatively small sample size.^33^ It is noticeable that overall the observed associations were more evident in older people (more than 65 years old) than in younger people. Older people with unintentional weight loss are more likely to have an unhealthy body composition, thus tend to have a higher risk of cancer. On the other hand, the unintentional weight loss in older people may also be an early warning sign for ESCC development.

In a prospective study exploring trajectory of perceived body shape across the lifespan in relation to all‐site cancer risk, body adiposity significantly increases the EAC risk in males,^20^ which is similar to the relationship between BMI and EAC risk. Our study indicates that perceived body shape at 20 years of age is not associated with ESCC incidence. However, individuals with ESCC tend to have a thinner perceived body shape at 10 years prior to ascertainment than controls. The change in perceived body shape from 20 years of age to 10 years prior to ascertainment demonstrates that a decrease in perceived body shape rating is associated with increased ESCC risk, and that an increase in perceived body shape rating is associated with reduced ESCC risk among participants who are thinner than perceived body shape 4. However, perceived body shape change was not significantly associated with ESCC risk among participants who were rated greater than perceived body shape 3. The outcome of perceived body shape change is similar to BMI status change in relation to the risk of ESCC, and the relationship between body size change and ESCC risk is confirmed in our study.

It is noteworthy that the risk patterns of body size at age 20 years and body size at 10 years prior to ascertainment for ESCC risk were different, which were consistent with previous reports.^14^, ^33^, ^34^ Martincorena et al^35^ recently reported that somatic mutations accumulated with age in normal esophageal mucosa, and cancer‐associated mutant clones in middle‐aged and elderly donors covered most areas of the epithelium. This implies that body size, a changeable exposure, measured at different time and its individual long‐term change have different effects for the somatic carcinogenic mutation or epigenetic modification. The trajectory of body size across the lifespan in relation to ESCC risk needs be further explored. Furthermore, with little prior knowledge, our study shows that individual BMI gain is associated with 50% decrease in the risk of ESCC only among individuals with underweight and normal weight in early adulthood, but such beneficial effects were not found among overweight or obese subjects in early adulthood.

While the possibility of effect modification by smoking and alcohol drinking on the inverse relationship between body size and ESCC risk has been explored and suggested in some studies,^14^, ^16^, ^29^, ^36^ we did not detect any joint effect of body size with sex, family wealth score, smoking, or alcohol drinking, which is consistent with results from several previous studies.^17^, ^33^, ^37^

The underlying biological mechanism of the association between height, BMI, or body shape and the risk of ESCC is not fully understood. Although height itself is not considered a carcinogenic factor, its apparent relationship with cancer risk may serve as a proxy for other exposures or risk factors. Albanes et al^38^ speculate that taller individuals may have an elevated risk of cancer due to higher cell turnover, steroid hormones, or growth factors, or may be at higher risk of malignant transformation due to the presence of greater numbers of cells. In our study, very lean body size was significantly associated with an increased risk of ESCC, especially in older people. Some studies have reported that lean body size may be related to poor diet leading to micronutrient deficiencies or malnutrition, which have been implicated as risk factors contributing to ESCC development.^39^, ^40^ However, the abnormal manifestations of immunity, metabolism and inflammation resulting from excess adiposity might promote the development of multiple cancers.^41^ The paradoxical protective effect of obesity in various chronic diseases, especially cancer survival, has been reported and explored in many studies, and the measurement of muscle and adiposity could reveal the obesity paradox and inform precision oncology care.^42^, ^43^ However, this information could not be collected in our case‐control study, and prospective studies are warranted to further explore the association between body size and the risk of ESCC.

Our study has several strengths. In order to decrease potential influence of recall bias, we interviewed most cases before they were aware of diagnosis. Other advantages of our study include the relatively large sample size, independent verification of cases, relatively high response rates among both cases and controls, and the systematic collection of lifelong and detailed data. It is noteworthy that we randomly selected controls from the local Population Registry.

Despite these strengths, the limitations of our study should also be mentioned. First, despite enormous efforts to recruit study subjects as completely as possible, there were still 20%‐30% nonrespondents. However, no significant differences were found between respondents and nonrespondents for age or gender distribution. Second, the information about muscle and adiposity prior to interview could not be obtained in our study. Moreover, body weight and perceived body shape data were not collected every 5 or 10 years. Current assessments only captured relative overall changes in body size. Finally, like other case‐control studies, recall bias may still exist despite the previously mentioned efforts to interview cases as early as possible. To address this, we performed a sensitivity analysis by excluding those cases identified solely from the local Cancer Registry who were interviewed after diagnosis. The similar results somewhat allay concerns for confounding related to recall bias.

In conclusion, we found that ESCC risk in adults rose sharply with increasing height up to 170 cm among men and 157 cm among women, and then plateaued. BMI and perceived body shape at distant years was not associated with ESCC incidence. However, BMI and perceived body shape 10 years prior to ascertainment showed a monotonic inverse linear relationship with ESCC risk. To the best of our knowledge, we are the first to show that individual body weight loss is associated with increased ESCC risk and individual body weight gain is associated with reduced ESCC risk among individuals with underweight and normal weight. Individual body weight change was not significantly associated with ESCC risk among overweight and obese individuals. These findings suggest that maintaining a fit body shape and a reasonable BMI is advisable and of vital importance to reduce the risk of ESCC, as well as to promote health in general. The potential biological mechanisms for these relationships need to be explored further in future studies.

## CONFLICT OF INTEREST

The authors declare that they have no potential conflicts of interest.
