# Supplementary figures and images for: Adult height, body mass index change, and body shape change in relation to esophageal squamous cell carcinoma risk: A population‐based case‐control study in China
Source: Cancer Med. 2019 Aug 1;8(12):5769–78. doi: 10.1002/cam4.2444 (PMC6746109; doi:10.1002/cam4.2444)

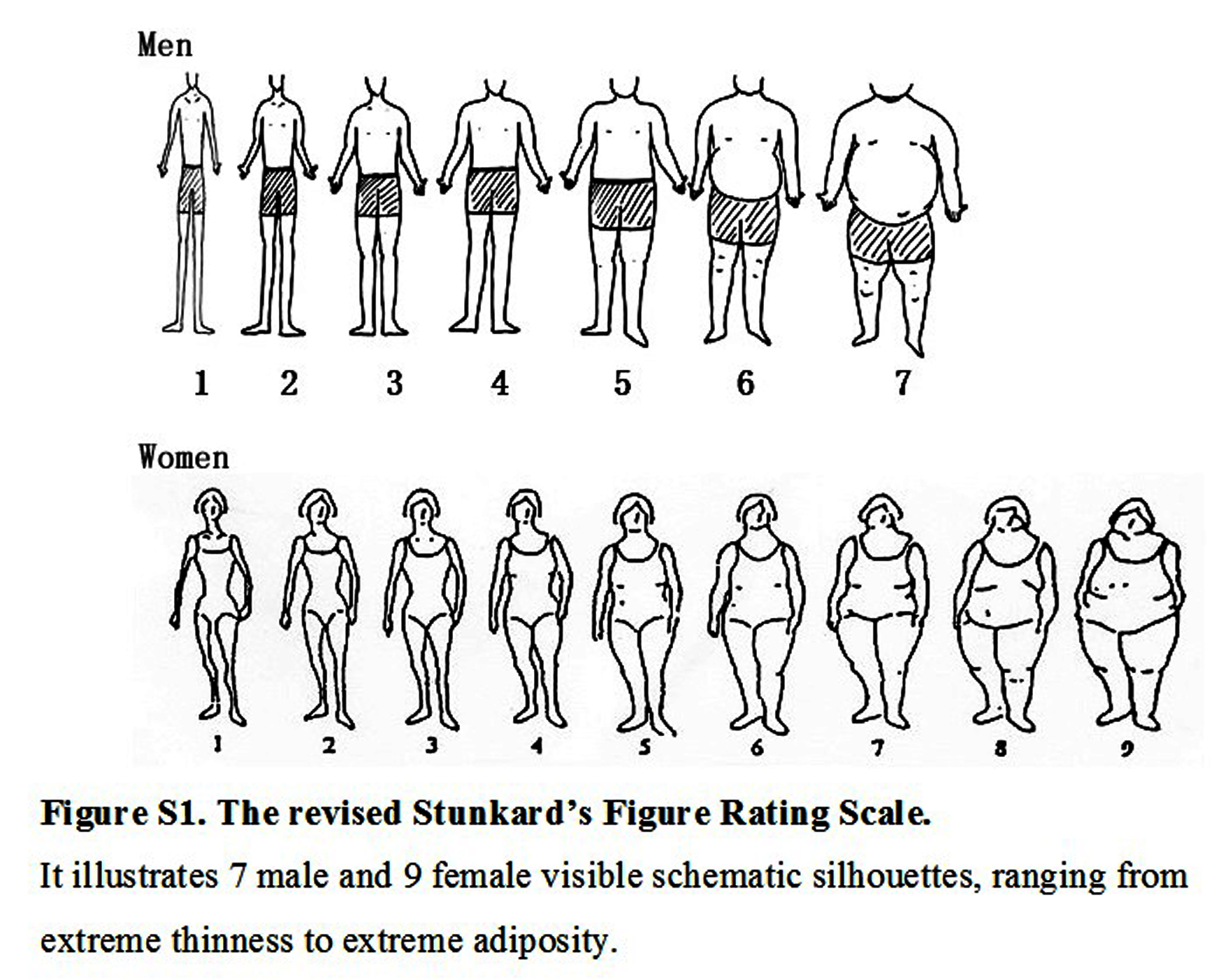

Supplement: Supplementary file 1 [file CAM4-8-5769-s001.tif]
