# Supplementary material for: Adult height, body mass index change, and body shape change in relation to esophageal squamous cell carcinoma risk: A population‐based case‐control study in China
Source: Cancer Med. 2019 Aug 1;8(12):5769–78. doi: 10.1002/cam4.2444 (PMC6746109; doi:10.1002/cam4.2444)
Supplement: Supplementary file 3 [file CAM4-8-5769-s003.docx]

| **Table S1.** Demographic Information of the Study Subjects Enrolled in a Population-based Case-control Study of Esophageal Squamous Cell Carcinoma, Taixing, China, 2010-2013. | | | |
| --- | --- | --- | --- |
| **Variables** | **Controls (N=1989)**  **N (%)** | **Cases**  **(N=1414)**  **N (%)** | ***P* value**^†^ |
| **Age at interview (mean±SD, years)** | 66.2 ± 8.8 | 66.6 ± 8.4 | 0.525 |
| **Sex** |  |  |  |
| Men | 1371 (68.93) | 959 (67.82) | 0.493 |
| Women | 618 (31.07) | 455 (32.18) |  |
| **Education level** |  |  |  |
| Illiteracy | 538 (27.05) | 497 (35.15) | <0.001 |
| Primary school | 759 (38.16) | 526 (37.20) |  |
| Junior high school | 530 (26.65) | 300 (21.22) |  |
| High school and above | 162 (8.14) | 91 (6.44) |  |
| **Marital status** |  |  |  |
| Unmarried | 67 (3.37) | 57 (4.03) | 0.216 |
| Married | 1587 (79.79) | 1094 (77.37) |  |
| Divorced/Widowed | 335 (16.84) | 263 (18.60) |  |
| **Occupation** |  |  |  |
| Farmer | 1257 (63.20) | 944 (66.76) | 0.857 |
| Worker | 401 (20.16) | 260 (18.39) |  |
| Service/Clerk/Professional/Administrator | 331 (16.64) | 210 (14.85) |  |
| **Family wealth score** |  |  |  |
| Q1-lowest | 408 (20.51) | 404 (28.57) | <0.001 |
| Q2 | 353 (17.75) | 255 (18.05) |  |
| Q3 | 434 (21.82) | 322 (22.77) |  |
| Q4 | 429 (21.57) | 262 (18.53) |  |
| Q5-highest | 365 (18.35) | 171 (12.09) |  |
| **Sum of missing and filled teeth** |  |  |  |
| None | 500 (25.14) | 304 (21.5) | 0.003 |
| < 6 | 717 (36.05) | 459 (32.46) |  |
| ≥ 6 | 749 (37.66) | 598 (42.29) |  |
| Missing | 23 (1.16) | 53 (3.75) |  |
| **Times of tooth brushing per day** |  |  |  |
| < 2 | 1290 (64.86) | 1110 (78.50) | ＜0.001 |
| ≥ 2 | 677 (34.04) | 261 (18.46) |  |
| Missing | 22 (1.11) | 43 (3.04) |  |
| **Tea temperature** |  |  |  |
| Never | 1435 (72.15) | 913 (64.57) | <0.001 |
| Warm | 225 (11.31) | 161 (11.39) |  |
| Hot | 221 (11.11) | 177 (12.52) |  |
| Very Hot | 80 (4.02) | 104 (7.36) |  |
| Missing | 28 (1.41) | 59 (4.17) |  |
| **Family history of esophageal cancer among first-degree relatives** |  |  |  |
| No | 1605 (80.69) | 928 (65.63) | ＜0.001 |
| Yes | 364 (18.30) | 439 (31.05) |  |
| Missing | 20 (1.01) | 47 (3.32) |  |
| **Smoking pack-years** |  |  |  |
| Never | 884 (44.44) | 566 (40.03) | 0.003 |
| ≤ 30 | 542 (27.25) | 342 (24.19) |  |
| > 30 | 535 (26.90) | 442 (31.26) |  |
| Missing | 28 (1.41) | 64 (4.53) |  |
| **Alcohol drinking intensity (g/day)** |  |  |  |
| Never | 1158 (58.22) | 635 (44.91) | <0.001 |
| ≤ 80 | 404 (20.31) | 320 (22.63) |  |
| > 80 | 398 (20.01) | 398 (28.15) |  |
| Missing | 29 (1.46) | 61 (4.31) |  |
| Abbreviations: SD, standard deviation; N, number.  ^†^ *P* values were derived using Wilcoxon rank-sum test for continuous variables and Chi-squared test for categorical variables, after excluding the corresponding missing value. | | | |

| **Table S2.** Association Between Height, BMI (at age 20 Years and 10 Years ago), Stunkard body shape (at age 20 Years and 10 Years ago) and Risk of Esophageal Squamous Cell Carcinoma, Taixing, China, 2010-2013. | | | | |
| --- | --- | --- | --- | --- |
| **Anthropometric measure** | **Controls**  **N (%)** | **Cases**  **N (%)** | **OR (95%CI )**^†^ | **OR (95%CI )**^‡^ |
| **Height (cm) in males** |  |  |  |  |
| 130-159 | 246 (17.94) | 53 (5.53) | 0.25 (0.18-0.36) | 0.23 (0.16-0.33) |
| 160-164 | 360 (26.26) | 149 (15.54) | 0.49 (0.38-0.63) | 0.44 (0.33-0.58) |
| 165-168 | 363 (26.48) | 305 (31.80) | 1.00 (Reference) | 1.00 (Reference) |
| 169-172 | 237 (17.29) | 252 (26.28) | 1.27 (1.00-1.60) | 1.31 (1.01-1.69) |
| 172-190 | 165 (12.04) | 200 (20.86) | 1.44 (1.12-1.87) | 1.55 (1.17-2.06) |
| *P* value for trend |  |  | <0.001 | <0.001 |
| Per 5 cm increment | 1371 (100) | 959 (100) | 1.46 (1.36-1.56) | 1.53 (1.42-1.66) |
| **Height (cm) in females** |  |  |  |  |
| 133-149 | 109 (17.64) | 14 (3.08) | 0.11 (0.06-0.21) | 0.11 (0.06-0.21) |
| 150-152 | 135 (21.84) | 52 (11.43) | 0.37 (0.24-0.55) | 0.31 (0.20-0.49) |
| 153-155 | 120 (19.42) | 126 (27.69) | 1.00 (Reference) | 1.00 (Reference) |
| 156-160 | 174 (28.16) | 180 (39.56) | 1.12 (0.80-1.56) | 1.14 (0.79-1.64) |
| 161-175 | 80 (12.94) | 83 (18.24) | 1.17 (0.78-1.75) | 1.48 (0.94-2.33) |
| *P* value for trend |  |  | <0.001 | <0.001 |
| Per 5 cm increment | 618 (100) | 455 (100) | 1.71 (1.52-1.93) | 1.90 (1.66-2.17) |
| **BMI status (kg/m^2^) at age 20 years** |  |  |  |  |
| < 18.5 (Underweight) | 221 (11.11) | 141 (9.97) | 0.88 (0.70-1.10) | 0.85 (0.66-1.08) |
| 18.5-24) (Normal) | 1343 (67.52) | 968 (68.46) | 1.00 (Reference) | 1.00 (Reference) |
| [24, 28) (Overweight) | 375 (18.85) | 269 (19.02) | 0.99 (0.83-1.18) | 0.98 (0.81-1.19) |
| ≥ 28 (Obese) | 50 (2.51) | 36 (2.55) | 0.98 (0.63-1.52) | 1.07 (0.66-1.73) |
| *P* value for trend |  |  | 0.566 | 0.379 |
| Per 5 kg/m^2^ increment | 1989 (100) | 1414 (100) | 1.09 (0.96-1.22) | 1.09 (0.96-1.25) |
| **BMI status (kg/m^2^) at 10 years ago** |  |  |  |  |
| < 18.5 (Underweight) | 110 (5.53) | 132 (9.34) | 1.58 (1.20-2.07) | 1.40 (1.04-1.87) |
| [18.5, 24) (Normal) | 1205 (60.58) | 903 (63.86) | 1.00 (Reference) | 1.00 (Reference) |
| [24, 28) (Overweight) | 545 (27.40) | 322 (22.77) | 0.79 (0.67-0.93) | 0.81 (0.68-0.97) |
| ≥ 28 (Obese) | 129 (6.49) | 57 (4.03) | 0.59 (0.42-0.82) | 0.63 (0.44-0.90) |
| *P* value for trend |  |  | <0.001 | <0.001 |
| Per 5 kg/m^2^ increment | 1989 (100) | 1414 (100) | 0.70 (0.63-0.79) | 0.73 (0.64-0.82) |
| **BMI change from age 20 years to 10 years ago (kg/m^2^)** |  |  |  |  |
| [-8,0) | 478 (24.03) | 527 (37.27) | 1.46 (1.21-1.76) | 1.50 (1.22-1.83) |
| 0 | 464 (23.33) | 350 (24.75) | 1.00 (Reference) | 1.00 (Reference) |
| (0,3] | 576 (28.96) | 303 (21.43) | 0.70 (0.57-0.85) | 0.73 (0.60-0.91) |
| (3,15] | 471 (23.65) | 234 (16.55) | 0.66 (0.53-0.81) | 0.70 (0.56-0.89) |
| *P* value for trend |  |  | <0.001 | <0.001 |
| BMI change per 1 kg/m^2^ increment |  |  | 0.91 (0.89-0.93) | 0.92 (0.89-0.94) |
| Abbreviations: BMI, body mass index; OR, odds ratio; CI, confidence intervals; N, number.  ^†^ Adjusted for age (continuous) and sex.  ^‡^ Adjusted for age (continuous), sex, marital status, education, occupation, family wealth score, sum of missing and filled teeth, number of tooth brushing per day, smoking pack-years, alcohol consumption intensity, tea temperature, and family history of esophageal cancer among first-degree relatives (except age, other variables are categorized as shown in Table S1). | | | | |
